# Meta-Analysis of the QTLome of Fusarium Head Blight Resistance in Bread Wheat: Refining the Current Puzzle

**DOI:** 10.3389/fpls.2019.00727

**Published:** 2019-06-13

**Authors:** Eduardo Venske, Railson Schreinert dos Santos, Daniel da Rosa Farias, Vianei Rother, Luciano Carlos da Maia, Camila Pegoraro, Antonio Costa de Oliveira

**Affiliations:** ^1^Crop Science Department, Plant Genomics and Breeding Center, Eliseu Maciel School of Agronomy, Federal University of Pelotas, Pelotas, Brazil; ^2^Instituto Federal de Educação, Ciência e Tecnologia Farroupilha (IFFar), Alegrete, Brazil; ^3^Instituto Federal de Educação, Ciência e Tecnologia Catarinense (IFC), Araquari, Brazil

**Keywords:** molecular markers, meta-QTL, genetic architecture, genetic maps, genome, transcriptome, bioinformatics

## Abstract

**Background:** Fusarium Head Blight (FHB) is a worldwide devastating disease of bread wheat (*Triticum aestivum* L.). Genetic resistance is the most effective way to control FHB and many QTL related to this trait have been mapped on the wheat genetic map. This information, however, must be refined to be more efficiently used in breeding programs and for the advance of the basic research. The objective of the present study was to in-depth analyze the QTLome of FHB resistance in bread wheat, further integrating genetic, genomic, and transcriptomic data, aiming to find candidate genes.

**Methods:** An exhaustive bibliographic review on 76 scientific papers was carried out collecting information about QTL related to FHB resistance mapped on bread wheat. A dense genetic consensus map with 572,862 loci was generated for QTL projection. Meta-analysis could be performed on 323 QTL. Candidate gene mining was carried out within the most refined loci, containing genes that were cross-validated with publicly available transcriptional expression data of wheat under *Fusarium* infection. Most highlighted genes were investigated for protein evidence.

**Results:** A total of 556 QTL were found in the literature, distributed on all sub-genomes and chromosomes of wheat. Meta-analysis generated 65 meta-QTL, and this refinement allows one to find markers more tightly linked to these regions. Candidate gene mining within the most refined meta-QTL, meta-QTL 1/chr. 3B, harvested 324 genes and transcriptional data cross-validated 10 of these genes, as responsive to FHB. One is of these genes encodes a Glycosiltransferase and the other encodes for a Cytochrome P450, and these such proteins have already been verified as being responsible for FHB resistance, but the remaining eight genes still have to be further studied, as promising loci for breeding.

**Conclusions:** The QTLome of FHB resistance in wheat was successfully assembled and a refinement in terms of number and length of loci was obtained. The integration of the QTLome with genomic and transcriptomic data has allowed for the discovery of promising candidate genes for use in breeding programs.

## Introduction

Fusarium Head Blight (FHB), also known as scab, is one of the most important diseases of bread or hexaploid wheat (*Triticum aestivum* L.), causing expressive losses every year in many countries (Buerstmayr et al., 2014; Steiner et al., 2017). It is a floral infection disease mainly caused by the fungus *Fusarium graminearum*, but also by *F. culmorum* and *F. avenaceum*, being highly influenced by the environment and it is difficult to control by chemical means (Parry et al., 1995; Bai and Shaner, 2004; Steiner et al., 2017). It is a serious problem for wheat production because it causes not only quantitative losses, reducing yield, but also qualitative damages, while the pathogen produces a set of mycotoxins, which are highly toxic to human and animals (Del Ponte et al., 2004; Draeger et al., 2007; Pestka, 2010). Among the most important mycotoxins, one can list Deoxynivalenol (DON) and Nivalenol (NIL), which are also pathogenicity factors (Draeger et al., 2007).

The most effective and convenient way to control this disease is through the adoption of resistant cultivars (Steiner et al., 2017). FHB resistance is a quantitative trait and it has been classically proposed and well-accepted that wheat can present five different types of resistance to this disease: viz., (I) to the initial infection of the pathogen within spike tissues; (II) to the subsequent spread of the disease throughout the spike; (III) to the accumulation of mycotoxins; (IV) to kernel infection and (V) to yield performance (Schroeder and Christensen, 1963; Miller and Arnison, 1986; Mesterhazy, 1995; Mesterhazy et al., 1999; Buerstmayr and Lemmens, 2015). Also, in some studies, resistance types I and II are assayed jointly, as at field conditions, i.e., without control regarding the inoculum presence and abundance, it is not possible to precisely determine the contribution of initial infection and fungal spread on each diseased head. Regarding methods to assay each of all these types of resistance, literature describes different protocols with this aim, from inoculation aspects to evaluation procedures, of which the descriptions, however, are not the main objective of the present study.

It has been recognized that wheat presents expressive genetic variability for FHB resistance (Steiner et al., 2017). Hundreds of QTL contributing to this trait were already mapped on the genetic map of the cereal, distributed evenly throughout all genomes and chromosomes of the species and verified in dozens of different cultivars and breeding lines (Buerstmayr et al., 2009; Kugler et al., 2013; Steiner et al., 2017). Despite of the expressive amount of QTL related to this trait mapped so far, there is a consensus among the researchers that only very few of these loci have in fact been utilized for the development of improved cultivars, resistant to this disease (Buerstmayr et al., 2014; Steiner et al., 2017).

In order to make these already mapped QTL more useful to plant breeding, as well as, for the basic research aiming to better understand FHB resistance in wheat and other small grain cereals, a comprehensive and in depth analysis of all these loci has to be carried out. However, due to the fact that most mapping experiments differ in numerous aspects, such as type of mapping population, number of lines, number, and type of molecular markers used and design and even the general quality of the experiment, an elaborated method has to be applied for this purpose. In this regard, QTL-meta analysis has been proven as an efficient approach, and has been constantly improved (Goffinet and Gerber, 2000; Salvi and Tuberosa, 2015).

In general, this analysis aims to verify whether QTL observed in isolated studies correspond to different loci or whether they actually represent a common position on the genetic map of the studied species, as well as the opposite i.e., if a QTL hitherto considered as mapped in a common region in diverse genotypes actually represents different positions. Finally, the analysis aims to stablish the occurrence of QTL “hotspots” in a consensus map, which correspond to the more precise region where these loci represent under analysis, the so-called “meta-QTL.” These refined genomic regions harbor important and, in many cases, several genes related to the trait (Goffinet and Gerber, 2000; Salvi and Tuberosa, 2015). This approach was already applied to many different crops and traits, such as cist nematode resistance in soybean (Guo et al., 2006), yield under drought conditions in rice (Swamy et al., 2011), diverse yield and quality traits in cotton (Said et al., 2015), yield in maize (Martinez et al., 2016) and yield, baking quality, and grain protein content related traits in wheat (Quraishi et al., 2017).

Three QTL meta-analysis studies were already performed for FHB resistance in wheat (Liu et al., 2009; Löffler et al., 2009; Mao et al., 2010—in chronological sequence). Among these studies, virtually simultaneous, one of special relevance was that published by Liu et al. (2009), who brought the largest amount of information generated until the year of 2009. Despite the comprehensiveness of the bibliographic review performed and the contribution in projecting distinct QTL in a common consensus map, the research could not take advantage of the current improved methods to determine meta-QTL. For the studies of Löffler et al. (2009) and Mao et al. (2010), they considered fewer QTL in the analyses, and the later focused specially in understanding the association between FHB resistance and plant height.

Many advances have been taking place that have currently improved algorithms but are still in constant evolution, and are available for QTL-meta analysis. Among these are the ones implemented in the software BioMercator (Arcade et al., 2004; Veyrieras et al., 2007; Sosnowski et al., 2012). These algorithms test, through the maximum likelihood method, the number of possible meta-QTL generated from the projection of distinct original QTL in a linkage group, and apply statistical criteria to suggest the best model to be used for deciding the actual number of these meta-loci (Goffinet and Gerber, 2000; Sosnowski et al., 2012).

The QTL meta-analysis for FHB resistance in wheat, carried out by Liu et al. (2009), Löffler et al. (2009), and Mao et al. (2010), utilized basically RFLP (Restriction Fragment Length Polymorphism) and SSR (Simple Sequence Repeat) markers. During and in the subsequent years after these studies, a revolution has taken place in terms of genotyping technology. Distinct technologies for SNP (Single Nucleotide Polymorphism) assessment have been developed (Akbari et al., 2006; Poland J. A. et al., 2012; Poland J. et al., 2012; Semagn et al., 2014; Wang et al., 2014; Winfield et al., 2016). From few RFLP or SSR loci by study, an expressive advance has taken place for the utilization of platforms which can evaluate thousands of loci at time, such as the Axiom® 820K SNP array, which can assess near to 820 thousand SNPs at once (Winfield et al., 2016). Furthermore, various studies in QTL mapping were performed since 2009 until now, thus there is an important accumulation of information to be treated or updated.

The other step-change, which was made recently in wheat research, was the completion of the genome sequence (International Wheat Genome Sequencing Consortium, 2014) and more recently, the genome annotation (International Wheat Genome Sequencing Consortium, 2018), which has been made freely available for the scientific community. In the same way, a large amount of transcriptomic data, including related to FHB response, has been made available, through a user-friendly platform (Borrill et al., 2016; Ramírez-González et al., 2018). All these resources represent a rich opportunity to unveil, in an in-depth way, the genetic architecture of FHB resistance in wheat.

Considering the exposed, the aim of this work was to perform a comprehensive meta-analysis of QTL related to FHB resistance in hexaploid wheat, further integrating genetic, genomic, and transcriptomic data, aiming to find promising candidate genes. It is expected that this work will contribute to both the improvement in strategies for wheat breeding for FHB resistance but also to the basic research aiming to better elucidate the genetic architecture underlying the trait.

## Materials and Methods

**Figure 1:**
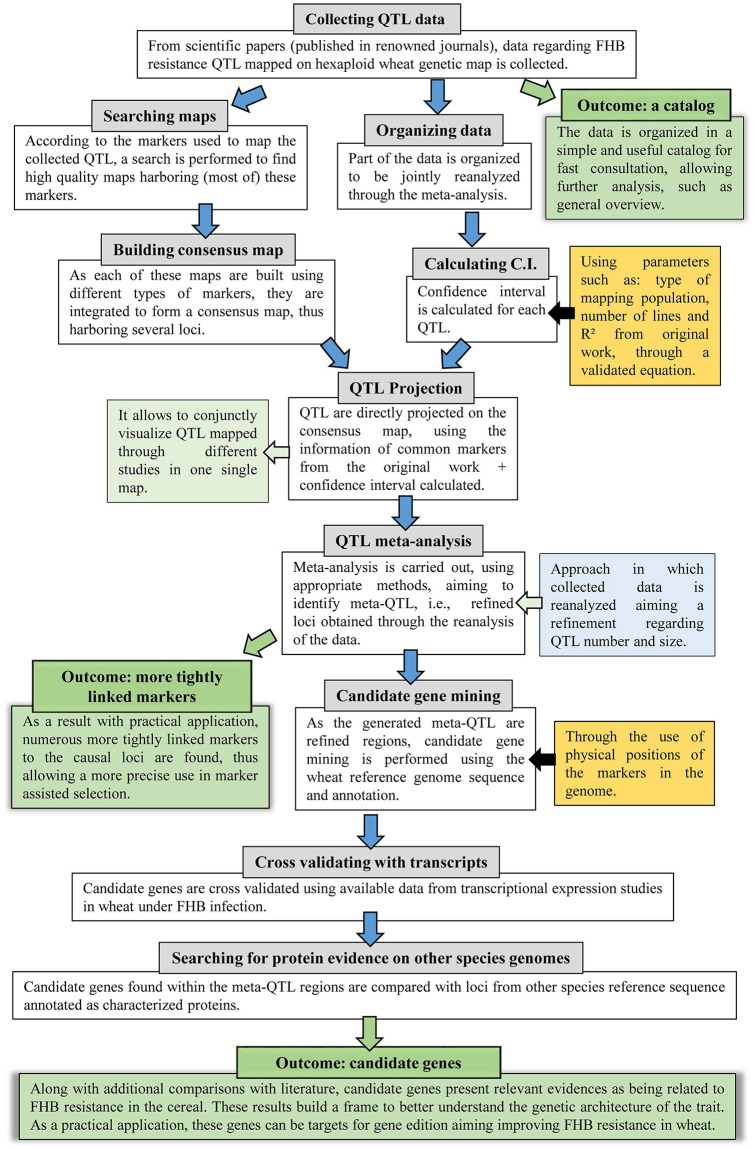
Flow diagram of the systematic review and QTL meta-analysis applied in this study, which further incorporated genomic and transcriptomic publicly available data.

The approach applied in this study comprises several steps, thus, to facilitate the understanding, a flow diagram is presented in Figure 1. Further description of each step is given as follows.

### Search Strategy—Collection and Characterization of QTL for FHB Resistance in Wheat

An exhaustive bibliographic review was carried out on 76 papers published between 1999 and 2017, searching for information on QTL mapped in bread wheat (*T. aestivum* L.) related to FHB resistance (Table 1, Supplementary File 1). These studies include the ones utilized in the meta-analysis performed by Liu et al. (2009), Löffler et al. (2009), and Mao et al. (2010).

**Table 1:** Publications from which the data was collected to form the database for this study.

**Year of publication**	**Number of papers**	**References**
1999	1	Waldron et al., 1999
2000	1	Gupta et al., 2000
2001	2	Anderson et al., 2001; Gupta et al., 2001
2002	2	Buerstmayr et al., 2002; Zhou et al., 2002
2003	6	Bourdoncle and Ohm, 2003; Buerstmayr et al., 2003; Gervais et al., 2003; Shen et al., 2003a,b; Somers et al., 2003
2004	6	Bai and Shaner, 2004; Lin et al., 2004; Paillard et al., 2004; Steiner et al., 2004; Zhang et al., 2004; Zhou et al., 2004
2005	5	Gilsinger et al., 2005; Jia et al., 2005; Mardi et al., 2005; Schmolke et al., 2005; Yang J. et al., 2005; Yang Z. et al., 2005
2006	7	Chen et al., 2006; Cuthbert et al., 2006; Lin et al., 2006; Liu et al., 2006; Ma et al., 2006a,b; Mardi et al., 2006
2007	10	Cuthbert et al., 2007; Draeger et al., 2007; Häberle et al., 2007; Jiang et al., 2007a,b; Klahr et al., 2007; Li et al., 2007; Liu et al., 2007; Semagn et al., 2007; Zhang and Mergoum, 2007
2008	7	Abate et al., 2008; Handa et al., 2008; Holzapfel et al., 2008; Malla et al., 2008a,b; Srinivasachary et al., 2008; Yu et al., 2008
2009	4	Bonin and Kolb, 2009; Häberle et al., 2009; Liu et al., 2009; Srinivasachary et al., 2009
2010	3	Malla et al., 2010; Zhang et al., 2010; Zhou et al., 2010
2011	3	Chu et al., 2011; Jayatilake et al., 2011; Li et al., 2011
2012	8	Basnet et al., 2012; Li et al., 2012; Liu et al., 2012; Lu et al., 2012; Suzuki et al., 2012; Szabó-Hevér et al., 2012; Zhang et al., 2012a,b
2013	1	Cativelli et al., 2013
2014	3	Cai and Bai, 2014; Chao et al., 2014; Szabó-Hevér et al., 2014
2015	2	Buerstmayr and Buerstmayr, 2015; Zhu et al., 2015
2016	4	He et al., 2016; Islam et al., 2016; McCartney et al., 2016; Petersen et al., 2016
2017	1	Petersen et al., 2017

For each QTL, the following information was collected: (1) type of resistance (Types I, II, I and II combined, III, IV, V), which are considered different traits; (2) genotype source of resistance, (3) type of the mapping population [(*F*_2_, backcross, recombinant inbreed lines (RILs) and double-haploids (DH)]; (4) number of assayed lines; (5) LOD (*logarithm of the odds*) score; (6) percentage of the phenotypic variance explained by the QTL (*R*^2^); (7) the flanking or single marker(s) (for interval mapping and single marker analysis, respectively). In the few cases in which the source-paper presented the *p-value* statistic or simply stated that a minimum LOD score of 3 was adopted to map the QTL, instead of informing the actual value, the LOD 3 was assumed in this study. Studies of association mapping or those using other non-common mapping populations were not included in this work.

### Consensus Map Development for QTL Projection

To project the diverse QTL collected, which were mapped using different types of markers, a consensus genetic map of wheat, resulting from the union of distinct individual maps, was developed, which was the basis for this study. The maps selected for the development of the consensus map, in a total of seven, were the ones available which harbor a large amount of the markers used for mapping the QTL for FHB resistance in wheat, whose information was obtained previously.

Thus, from GrainGenes website (https://wheat.pw.usda.gov/GG3/), the following maps were obtained: “*Wheat, Consensus SSR, 2004*,” “*Wheat, Composite, 2004*” e “*Wheat, Synthetic x Opata, BARC*,” as a source of SSR markers and few classical genes, such as *Vrn1*—a gene for vernalization sensitivity, which have also been used for QTL mapping in some studies. The “*Wheat consensus map version 4.0*,” source of DArT markers was obtained from the website of the company owner of this technology (https://www.diversityarrays.com). For chip-based SNP markers, assessed by the platforms *Illumina*^®^ *9K iSelect Beadchip Assay* and *Illumina*^®^ *iSelect 90K SNP Assay*, the maps were obtained from Cavanagh et al. (2013) and Wang et al. (2014), respectively. Finally, for SNPs obtained and mapped through the GBS (genotyping by sequencing) approach, the map of Saintenac et al. (2013) was used. With the exception of “*Wheat, Synthetic x Opata, BARC*,” all remaining maps are already consensus maps between different populations, thus they represent high quality maps. For the construction of the consensus map, the R package LPMerge was used (Endelman and Plomion, 2014).

### Projection and QTL Meta-Analysis

From the 556 QTL collected, 359 could be projected onto the consensus map generated, given that the markers used to map them were present in the consensus map. However, even QTL which could not be projected or analyzed via the meta-analysis were still collected and characterized, being present in a catalog with all information collected in this study (Supplementary File 1).

For the projection of these QTL, initially the confidence interval (CI) of 95% was calculated for each locus, through the equations below, which have been modeled for each mapping population (Darvasi and Soller, 1997; Guo et al., 2006): F2 and Backcross:

$$CI\;=\;\left(\frac{530}{(Number\;of\;lines\;x\;R^{2})\;}\right)\;$$

RILs:

$$CI\;=\;\left(\frac{163}{(Number\;of\;lines\;x\;R^{2})\;}\right)\;$$

Double-haploids:

$$CI\;=\;\left(\frac{287}{(Number\;of\;lines\;x\;R^{2})\;}\right)\;$$

Next, using the software BioMercator V4.2 (Arcade et al., 2004; Sosnowski et al., 2012), the QTL, represented by their middle points, along with their calculated confidence intervals, original LOD score and *R*^2^, were directly projected onto the consensus map previously developed, and the meta-analysis was carried out, individually by chromosome, through the *Veyrieras* two-step algorithm in the software (Veyrieras et al., 2007). The Akaike (AIC) statistics was used to define the best model for the definition of the number of meta-QTL or “real” QTL, which best represent the original QTL. The algorithms and statistical procedures implemented in this software are well-described in the literature (Arcade et al., 2004; Veyrieras et al., 2007; Sosnowski et al., 2012). As a requirement of the method, the meta-analysis could only be performed with linkage-groups with a minimum of 10 projected QTL. Attempts to run analysis when <10 QTL were projected returned in error. Thus, 323 QTL could, in fact, be considered for the QTL meta-analysis. All files prepared to run BioMercator V.4.2, i.e., maps and QTL files for each wheat chromosome, are made available in Supplementary File 2.

### Candidate Gene Mining, Transcriptional Expression Analysis, and Functional Annotation Within Meta-QTL

Among the meta-QTL obtained, the ones considered highly reliable and refined were considered for gene mining. The criteria adopted were as follows: (1) the meta-QTL is generated through the projection of at least two overlapping QTL (otherwise the meta-QTL would simply correspond to a single QTL previously known); (2) it is shorter than 1.0 cM in genetic distance; (3) it is shorter than 20 Mb in physical distance (at Chinese Spring wheat reference genome). For that, the physical positions of markers present at the flanking regions of each highly refined meta-QTL (corresponding to the end of the confidence interval) were searched at the International Wheat Genome Sequencing Consortium reference genome sequence repository at https://wheat-urgi.versailles.inra.fr/Seq-Repository/BLAST through BLAST search of their sequences or either directly through surveying the annotation browser (International Wheat Genome Sequencing Consortium, 2014, 2018). The “IWGSC RefSeq v1.0” version was used. High confidence annotated genes (*HighConfidenceGenesv1.1*), within each highly refined meta-QTL, were then listed and thereafter called candidate genes.

For the transcriptional expression analysis, the collected candidate genes were analyzed through the *expVIP* (expression Visualization and Integration Platform)/*Wheat Expression Browser* online resource (http://www.wheat-expression.com) (Borrill et al., 2016; Ramírez-González et al., 2018). The available data for “*Fusarium head blight infected spikelets*” (Kugler et al., 2013) was used. This study assayed the transcriptome profile of a set of wheat lines after *F. graminearum* point inoculation and control conditions (mock) at 30 and 50 h after treatment. The tissue analyzed was spikelets. From this data set, only transcripts from the varieties, CM-82036 (resistant to FHB) and a susceptible near isogenic line, were selected (a cross between CM-82036 and the susceptible variety Remus). Differentially expressed genes (considering disease × mock conditions, only) were collected, based on the standard error. Following the proposed criteria by Wagner et al. (2013), only genes presenting at least 2 transcripts per million (TPM) were considered in this search. Other transcriptomic studies approaching other sources of FHB resistance could not be used in this work as they were not made available in manageable platforms like the one we have used here. Next, differentially expressed genes were further investigated for functional annotation (i.e., protein evidence), also at the International Wheat Genome Sequencing Consortium reference genome sequence repository (https://wheat-urgi.versailles.inra.fr/Seq-Repository/BLAST).

## Results

### Characterization of QTL for FHB Resistance in Wheat

**Figure 2:**
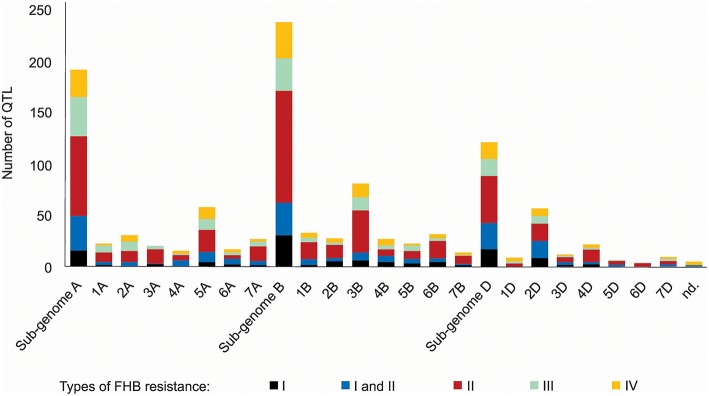
QTL related to FHB resistance in wheat published from 1999 to 2017, classified according the type of resistance they confer and the sub-genome and chromosome of occurrence.

A thorough search in the literature resulted in the collection of information regarding 556 QTL described as responsive for FHB resistance, already mapped on a bread wheat genetic map, distributed on all wheat sub-genomes and chromosomes (Figure 2). The wheat B sub-genome had the largest number of QTL mapped, with 238, followed by A, with 192 and finally D, with 121 QTL. The chromosomes 3B (81 QTL), 5A (58 QTL), and 2D (57 QTL) were the ones which presented the largest amount of these mapped regions. The most common type of resistance mapped was the type II (spread of the disease throughout the spike), with 41.5%, and the less mapped trait was the type I (initial infection), with 11.5% of the collected QTL. No QTL related to type V resistance was found, the resistance type corresponding to the ability of the genotype to tolerate the pathogen without losing yield potential. Five QTL were not assigned in the original study (Szabó-Hevér et al., 2014) to any wheat sub-genome or chromosome. The QTL collected were mapped from 77 different genotypes (cultivars or breeding lines), developed in many different countries (Supplementary File 1). Also, some of these loci were verified as belonging to the susceptible or moderately susceptible parent of the cross, which generated the mapping population, e.g., as in Cai and Bai (2014).

### Generation of the Consensus Map

Seven wheat genetic maps were used for the development of the consensus map, the base for the QTL projection and meta-analysis performed in this study. The generated map was composed by SSR, DArT, SNPs (from different technologies) and few genes, presenting 572,862 loci in total. On average, each chromosome presented 27,279 markers and was 217.49 cM long (Table 2). Attempts to incorporate more maps, containing thousands of other loci, aiming to form an even larger consensus map, were performed, however numerous ordering conflicts appeared. Thus, the maps integrated in this study presented the best harmonious combination. The consensus map, separated by chromosome (or linkage group), is available through Supplementary File 2.

**Table 2:** Consensus genetic map of wheat, generated from different maps and composed by SSR, DArT, SNP molecular markers and few genes.

**Linkage group (chromosome)**	**Consensus map**	
	**Length (cM)**	**Number of markers**
1A	255.6	24,278
1B	288.57	39,996
1D	208.05	16,029
2A	251.3	27,131
2B	316.13	47,062
2D	166.17	29,300
3A	207.27	23,619
3B	204.28	44,087
3D	188.33	21,470
4A	272.1	29,584
4B	135.76	18,828
4D	170.43	8,461
5A	274.91	28,558
5B	226.9	34,023
5D	223.97	15,264
6A	183.44	24,656
6B	134.46	31,794
6D	203	16,482
7A	226.6	33,174
7B	188.64	38,038
7D	241.29	21,028
Average	217.49	27,279
Total	4567.2	572,862

### QTL Projection and Meta-Analysis

**Figure 3:**
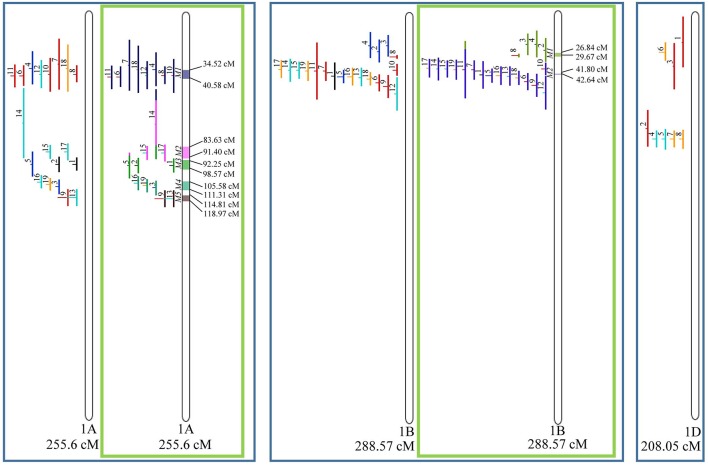
Projection (left) and, with green borders meta-QTL of FHB resistance in wheat for chr. 1A, 1B, and 1D. In the projection, the colors of the QTL means: black, type I resistance; red, type II; blue, type I and II combined; green, type III; orange, type IV. In the meta-analysis, the colors both in the QTL and inside the linkage groups only mean the different meta-QTL generated and how each QTL contributed to the formation of it. Numbering of the meta-QTL is located to the left side of the linkage group and to the right side is indicated the genetic distance which comprise these loci. The molecular markers and their positions are omitted in the figures for better visualization. The line in the middle of each QTL represents the LOD score of it in the original work.

**Figure 4:**
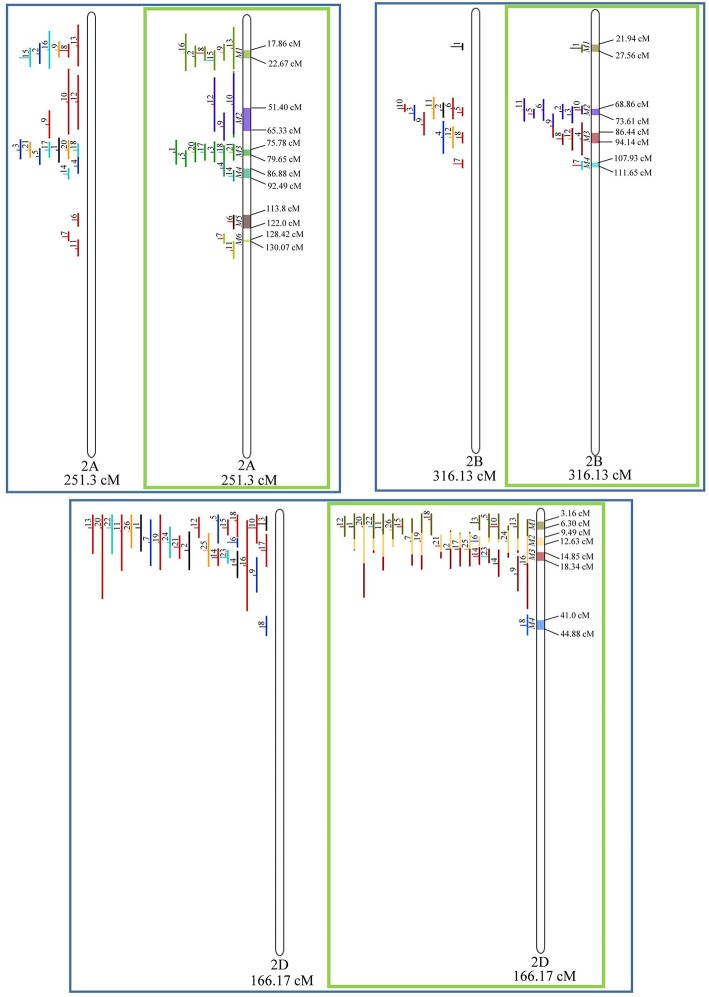
Projection (left) and, with green borders meta-QTL of FHB resistance in wheat for chr. 2A, 2B, and 2D. The color system and numbering follows the same as Figure 3.

**Figure 5:**
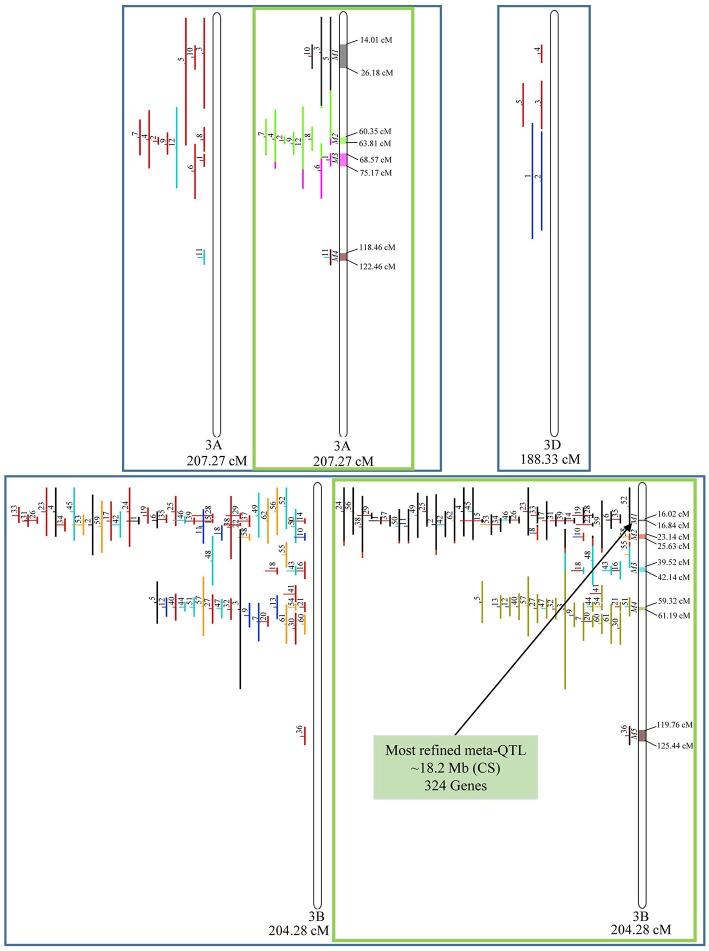
Projection (left) and, with green borders meta-QTL of FHB resistance in wheat for chr. 3A, 3B, and 3D. The color system follows the same as Figure 3. The meta-QTL 1 in 3B was selected for gene mining.

**Figure 6:**
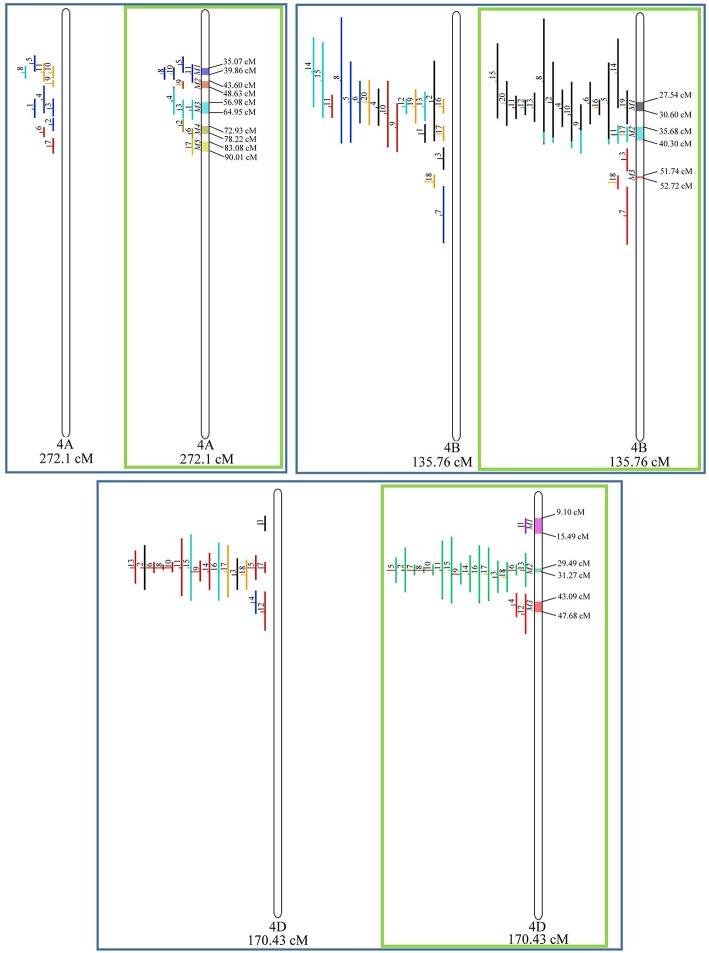
Projection (left) and, with green borders meta-QTL of FHB resistance in wheat for chr. 4A, 4B, and 4D. The color system follows the same as Figure 3.

**Figure 7:**
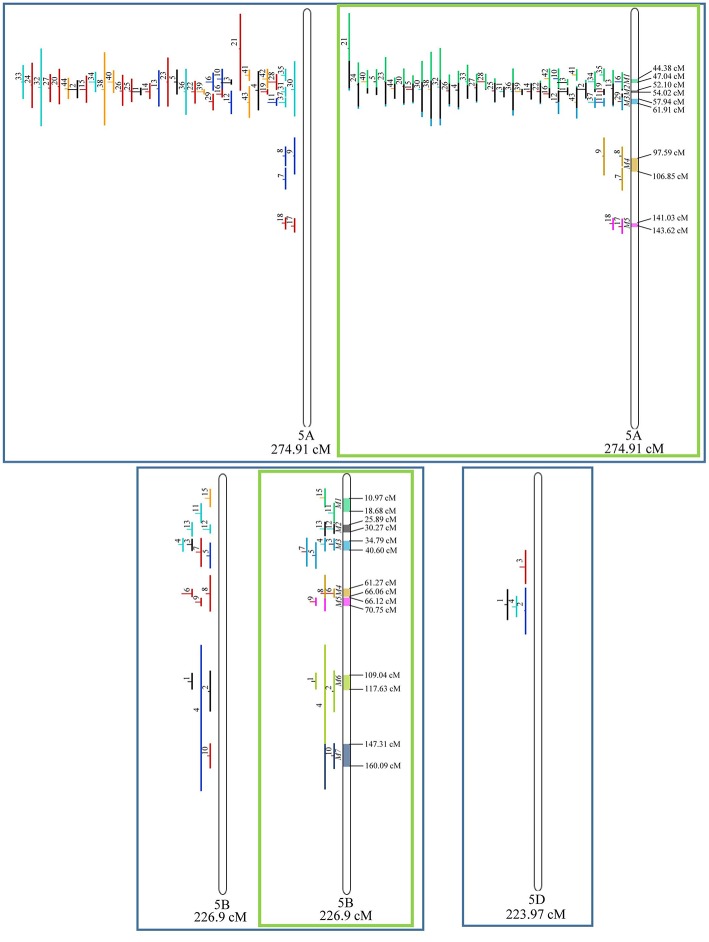
Projection (left) and, with green borders meta-QTL of FHB resistance in wheat for chr. 5A, 5B, and 5D. The color system follows the same as Figure 3.

**Figure 8:**
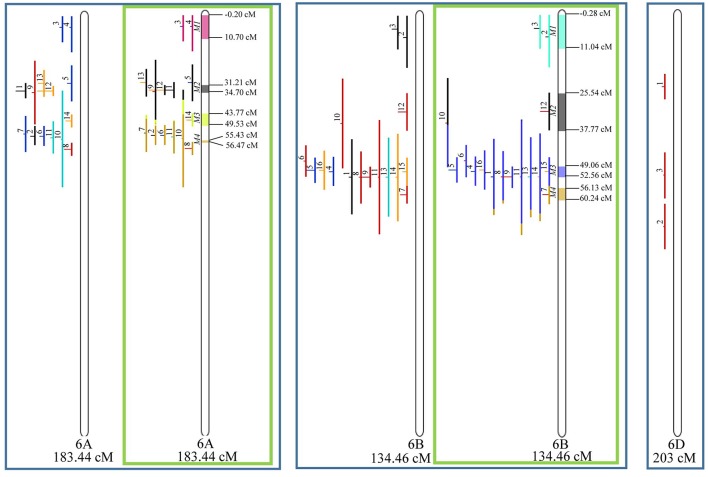
Projection (left) and, with green borders meta-QTL of FHB resistance in wheat for chr. 6A, 6B, and 6D (only projection). The color system follows the same as Figure 3.

**Figure 9:**
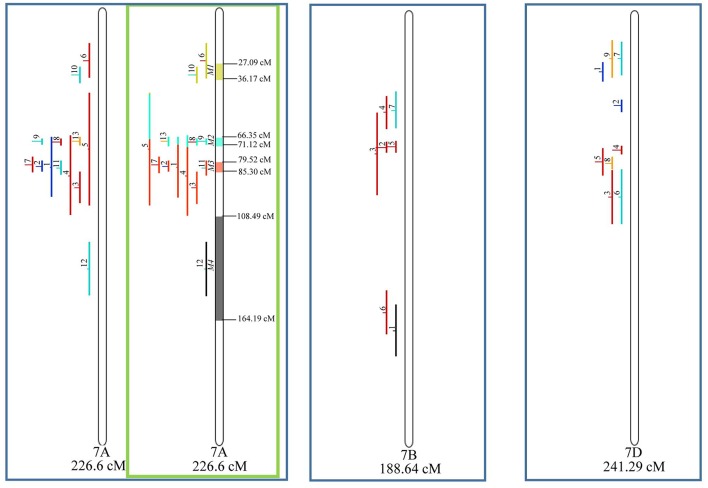
Projection (left) and, with green borders meta-QTL of FHB resistance in wheat for chr. 7A, and only projection for 7B and 7D. The color system follows the same as Figure 3.

From the 556 QTL collected from literature, a total of 359 could be projected on the consensus genetic map generated (Figures 3–**9**). It was decided not to include in the meta-analysis QTL mapped with the aid of AFLP and RFLP markers, as they usually originate from less dense maps, leading to large QTL. It could impair the attempt to refine the QTL regions. The remaining QTL which could not be included in the meta-analysis were due to the absence of their flanking markers in the consensus map developed.

Given that the method applied in this work requires the minimum of 10 QTL projected on a linkage group (chromosome) to launch a meta-analysis, it was possible to run the analysis in 15 out of the 21 linkage groups of hexaploid wheat. Thus, from the 359 QTL projected, 323 definitely composed the database for meta-QTL discovery. As a result of the meta-analysis, these 323 QTL gave origin to 65 meta-QTL, from which 33 were localized on the A sub-genome, 25 on the B sub-genome and 7 on the D sub-genome. The genetic length of these loci ranged from 0.82 to 55.7 cM with an average of 6.08 cM. For 1D, 3D, 5D, 6D, 7B, and 7D, the number of QTL projected (<10) was insufficient to perform the meta-analysis. In addition to the graphical form, which is presented here, all meta-QTL generated are available in Supplementary File 3, projected onto the genetic map, along with all markers comprising their entire length.

In chromosome 1A, 19 QTL were projected, and gave origin to 5 meta-QTL. In 1B, on the other hand, 19 QTL were also projected, however, only 2 meta-QTL were discovered, which was the smallest number of these refined loci obtained per chromosome, among all chromosomes that could undergo this analysis (Figure 3). For 1D, although QTL meta-analysis could not be performed, it is possible to verify that 5 out of the 8 projected loci co-localized in the linkage group. The homeologous group 2 (chr 2A, 2B, and 2D) was the one where the largest number of meta-QTL were verified (Figure 4). In chr 2A, from 22 QTL, six meta-QTL were calculated, and for 2B and 2D, four meta-QTL were found from each of the analyses of 12 and 26 projected QTL, respectively. In chr. 3A, 4 meta-QTL were detected from 12 projected QTL, but in 3D only the projection of 5 QTL could be performed (Figure 5). It was in chr. 3B where the largest amount of QTL was projected in this study, in a total of 62, which gave origin to 5 meta-QTL.

Similarly, to which was verified for the homeologous group 2, it was possible to perform the meta-analysis on all 3 chr. of the group 4, being 5, 3, and 3 meta-QTL verified in 4A, 4B, and 4D, respectively (Figure 6). Regarding the group 5, chr. 5A was the second in this work in terms of number of QTL projected, in a total of 44, which allowed the detection of 5 meta-QTL (Figure 7). For chr. 5B, seven meta-QTL were obtained, the largest number among all wheat chromosomes, however for 5D, it was only possible to do the projection of 4 QTL. Chromosomes 6A and 6B presented 4 meta-QTL each, while for 6D, the projection of only three QTL was possible, which was the smallest number of QTL projected in a linkage group in this study (Figure 8). The homologous group 7 was the one in which the smallest number of QTL were projected and the smallest number of meta-QTL were obtained, as the meta-analysis could only be carried out for 7A and 4 meta-QTL were found (Figure 9). As a special feature in this linkage group, the projection of one QTL (QTL 12) with an imprecise confidence interval led to the formation of one meta-QTL (meta-QTL 4/7A) even larger than the original QTL, being the largest meta-QTL verified in this study (55.7 cM).

### Candidate Gene Mining, Transcriptional Expression Within Meta-QTL, and Functional Annotation

Among the 65 meta-QTL obtained, only one fulfilled the previously established criteria for candidate gene mining, i.e., simultaneously resulting from the analysis of at least two overlapping QTL, being shorter than 1.0 cM in genetic length and also shorter than 20 Mb in physical length (based on the wheat genome reference sequence of Chinese Spring), which was the meta-QTL 1 of chromosome 3B. This meta-QTL presented the narrowest length in genetic distance among all 65 meta-QTL verified, i.e., 0.82 cM, which corresponded to 18.2 Mb in physical distance.

**Figure 10:**
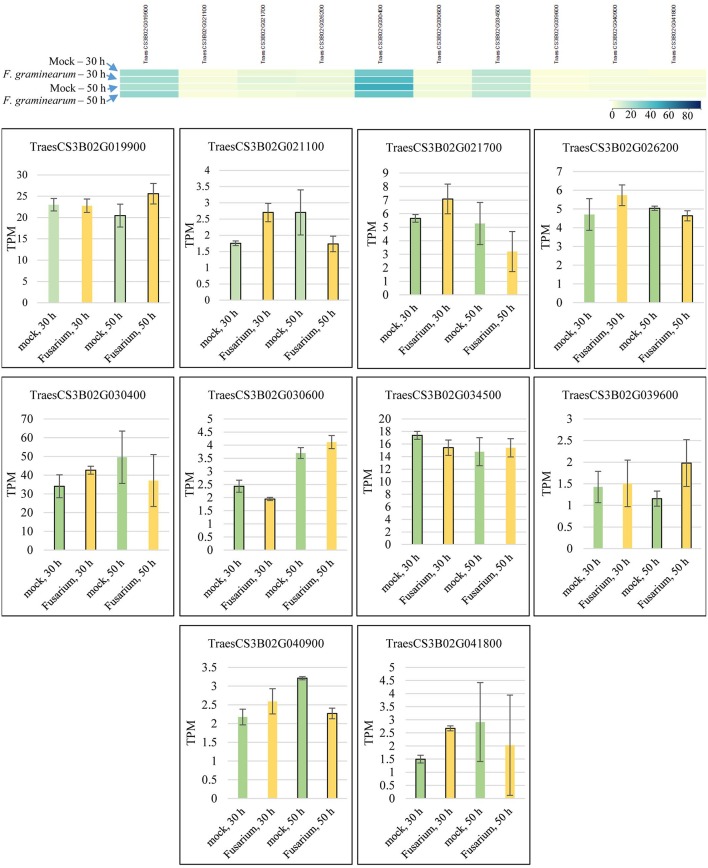
Transcriptional expression of candidate genes mined within the meta-QTL 1/3B, using publically available transcriptional data of wheat spike tissues (Kugler et al., 2013), after *Fusarium graminearum* inoculation and control (mock) conditions in the resistant variety CM-82036. TPM, transcripts per million.

Candidate gene mining within meta-QTL 1/3B, using the annotation of wheat reference genome sequence of Chinese Spring, resulted in a list of 324 genes (Supplementary File 4). These genes were investigated regarding expression under control and FHB occurrence, using publicly available data (Kugler et al., 2013). A total of 10 genes were found as differentially expressed in the resistant variety CM-82036 (Figure 10). None of these genes were differentially expressed in the susceptible line assayed. From these genes, five were differentially expressed only at 30 h after inoculation, four only at 50 h after inoculation, and one at both periods. Also, five genes were up-regulated when challenged by the pathogen, four down-regulated, and one up-regulated at 30 h and down-regulated at 50 h after inoculation.

The genes were further searched for functional annotation (protein evidence) analysis (Table 3). Five genes were only found as either uncharacterized, putative, or predicted proteins. From the five remaining genes, the first has been annotated as a Sarcoplasmic reticulum histidine-rich calcium-binding protein, in *Zea mays*, the second as a Glycosyltransferase, again in *Z. mays*, the third a Cytochrome P450 in *Aegilops tauschii, Oryza sativa* subsp. *japonica*, and *Triticum Urartu* and a Transposon protein in *Z. mays*, the fourth a Nuclease PA3, in *Z. mays*, and finally, the last a Metal tolerance protein, in *O. sativa* subsp. *japonica, Triticum Urartu*, and *Z. mays*.

**Table 3:** Protein evidence for the candidate genes mined within Meta-QTL 1 of chromosome 3B and differentially expressed after *Fusarium graminearum* inoculation in the variety CM-82036 (Kugler et al., 2013).

**Gene ID**	**Annotation at IWGSC—Protein evidence from other species**
TraesCS3B02G019900	Sarcoplasmic reticulum histidine-rich calcium-binding protein (*Zea mays*).
TraesCS3B02G021100	Annotated only as uncharacterized, predicted or putative protein.
TraesCS3B02G021700	Glycosyltransferase (*Zea mays*).
TraesCS3B02G026200	Cytochrome P450 (*Aegilops tauschii, Oryza sativa* subsp. *japonica, Triticum urartu*), Transposon protein (*Zea mays*).
TraesCS3B02G030400	Annotated only as uncharacterized protein.
TraesCS3B02G030600	Annotated only as uncharacterized protein.
TraesCS3B02G034500	Annotated only as uncharacterized protein.
TraesCS3B02G039600	Nuclease PA3 (*Zea mays*).
TraesCS3B02G040900	Metal tolerance protein (*Oryza sativa* subsp. *japonica, Triticum urartu, Zea mays*).
TraesCS3B02G041800	Annotated only as uncharacterized protein.

## Discussion

During the last two decades, starting with the first studies published by Bai et al. (1999) and Waldron et al. (1999) until today, several papers have been published on QTL mapping for FHB resistance in bread wheat (Steiner et al., 2017). It has motivated the execution of the present work, in which we attempted to re-analyze all these loci jointly, aiming to refine this information in order to be better used by breeders and also for the advance of the basic research. To the best of our knowledge, this is the largest collection and meta-analysis of QTL related to FHB resistance in bread wheat performed so far, and one of the largest of this kind already carried out for this cereal. The totality of QTL here analyzed can receive the term, recently proposed, of “QTLome” (Salvi and Tuberosa, 2015; Martinez et al., 2016) of FHB resistance in wheat, as it assembles in a global analysis most of the more important loci mapped in the crop until now. We have here updated the QTL-meta analysis previously performed by Liu et al. (2009), Löffler et al. (2009), and Mao et al. (2010), but more than that, we have applied the current improved algorithms and software, not available at the time these studies were carried out. Moreover, we have integrated genomic and transcriptomic data, which were also a recent resource for wheat research.

A total of 556 QTL were collected from the literature as being responsible for FHB resistance in wheat. This is a considerably large number; however, some level of redundancy was expected within this group of loci, which is why the meta-analysis was necessary. A total of 323 QTL could be included in the meta-analysis and generated 65 meta-QTL, i.e., regions statistically validated as unique. Although the redundancy was decreased by roughly five times, the resulting number of refined loci can still be considered as large. Just to compare, another recent study of this kind performed on hexaploid wheat has found a total of 32 meta-QTL, but by combining yield, baking quality and grain protein traits (Quraishi et al., 2017). The large number of meta-QTL obtained in this study emphasizes the wealth of genetic variability present in wheat gene pool for FHB resistance, as already recognized by other researchers (Steiner et al., 2017). Interestingly, it does not agree with the fact that hexaploid wheat has been considered as a species with narrow genetic variability, an issue that has supposedly been aggravated by modern breeding practices (Fu and Somers, 2009; Charmet, 2011).

The refinement obtained in this study was variable among loci. In some situations, such as for meta-QTL 1/3B, a total of 36 QTL (whole QTL or parts) were clustered in one single locus, which is a high reduction of redundancy. On the other hand, meta-QTL 5/3B was the result of the analysis of only one original QTL. Furthermore, in six linkage groups, the meta-analysis could not be performed, due to the insufficient number of QTL projected. For these under-represented loci, more QTL have to be added to the analysis to validate these regions as responsible for the trait. Even after several years of research in the search of QTL related to FHB resistance in wheat, the reports continue in an elevated rate (Steiner et al., 2017). Just to exemplify, during the final writing of this paper, Silva et al. (2019) have mapped another four QTL for FHB resistance and DON content. However, a decisive improvement in QTL meta-analysis would be the integration of data also from genome wide association studies (GWAS), however the available methods and algorithms still do not deal with such. Through GWAS, several genotypes are assayed, so several loci can be mapped in a single study. Also, the method allows for more precision in QTL mapping, which, if joined in a meta-analysis with QTL obtained from biparental mapping population, could harvest more refined meta-QTL. In fact, important GWAS have been carried out for FHB resistance in wheat (Kollers et al., 2013; Arruda et al., 2016).

When collecting the data to carry out this work, information about QTL conferring the five different types of FHB resistance were collected. All these QTL, which represent, in fact, distinct traits, were analyzed simultaneously, as responsible by the single “meta-trait” FHB resistance. It can be verified that the majority of the meta-QTL generated throughout this study were composed by the projection of QTL related to different types of resistance to this disease. Although this classification of types of FHB resistance is broadly accepted among researchers (Steiner et al., 2017), it is also acceptable that both similar mechanisms could play a role in each type of resistance as well as that within each of these mapped QTL, therefore genes for different traits can exist. In fact, the most important locus for FHB resistance already studied, *Fhb1*, has been confirmed as conferring resistance to the spread of the disease (type II resistance) as well as to the mycotoxin accumulation (type III resistance) (Lemmens et al., 2005; Gunupuru et al., 2018).

Meta-QTL are possibly genomic regions highly rich in genes. For maize, it was already deeply elucidated, in which the QTLome for yield potential presented a high correlation with gene density in the genome of the species (Martinez et al., 2016). In rice, within each meta-QTL detected for drought tolerance, candidate genes were mined, through comparative genomic approaches, as most of these genes were already suggested as being related to the trait, according to previous studies (Swamy et al., 2011). For wheat, within 32 meta-QTL related to yield, baking quality and grain protein content, a total of 15,772 genes were listed, but only 37 were considered as major candidates (Quraishi et al., 2017). In the present study, gene mining was only carried out in 1 meta-QTL (meta-QTL 1/3B), in which 324 candidate genes were listed, with only 10 cross validated as differentially expressed in CM-82036 under *F. graminearum* infection.

The most important FHB resistance locus studied so far, namely the QTL *Fhb1*, firstly mapped in the Chinese cultivar Sumai 3, is localized in the short arm of the 3B chromosome (Cuthbert et al., 2006; Bernardo et al., 2012; Gunnaiah et al., 2012; Rawat et al., 2016). In this study, the genetic position of this locus corresponds to meta-QTL 1 of the chromosome 3B. Although still in dispute, a recent study has suggested that *Fhb1* from Sumai 3, harbors as the main candidate for the trait expression, a gene which encodes a chimeric lectin with agglutinin domains and a pore-forming toxin-like domain (Rawat et al., 2016). In this study, it was not expected to find this gene within meta-QTL 1/3B, since we have used the sequence and annotation of Chinese Spring as a reference genome, which has been proven not to harbor an active copy of the gene (Rawat et al., 2016; Schweiger et al., 2016). Thus, since the gene evidenced by Rawat et al. (2016) is not present in meta-QTL 1/3B, all other genes found as differentially expressed within this meta-QTL could also be suggested as promising candidate genes, even for minor effect. It is important to mention that in order to examine the transcriptional expression of the listed candidate genes, we have used the available data for CM-82036, a variety derived from Sumai-3 and which harbors *Fhb1*. Both Sumai-3 and CM-82036 were part of the donors of QTL, which composed the analysis carried out in this study.

Five out of the 10 candidate genes which showed differential expression when CM-82036 was challenged by *F. graminearum* are annotated only as uncharacterized, putative or predicted proteins. These genes deserve further studies, in order to unveil their possible role in FHB resistance and future use in breeding programs. Regarding the differentially expressed genes annotated as Sarcoplasmic reticulum histidine-rich calcium-binding protein and metal tolerance protein, these genes have also been verified in other transcriptome studies, which also assayed Sumai-3 derived lines (Hofstad et al., 2016; Schweiger et al., 2016). To the best of our knowledge, nuclease PA3 and transposon proteins have not been attributed to FHB resistance mechanisms in wheat so far.

Glycosyltransferases play a vast number of roles in living organisms and have already been attributed to FHB resistance mechanisms in wheat, especially the uridinediphosphate glycosyltransferases (UDP-Glycosiltransferases) (Li et al., 2015; Xin et al., 2015; He et al., 2018). The main role of this molecule would be the Deoxynivalenol (DON) inactivation, and DON is a *F. graminearum* pathogenicity factor. In the transcriptome dataset we have explored, this gene was up-regulated when CM-82036 was challenged by the pathogen (Kugler et al., 2013). Regarding Cytochrome P450, previous studies have also shown the role of these proteins on wheat defense against *Fusarium* infection and DON accumulation. In Sumai 3 and CM-82036, the higher expression of this gene has been considered as a resistance mechanism, potentially involved in the biosynthesis of the wheat defense compound, DIMBOA (2,4-dihydroxy-7-methoxy-1,4-benzoxazin-3-one), which is known to be toxic to *F. graminearum* (Li et al., 2010; revised by Gunupuru et al., 2018; Kazan and Gardiner (2018). However, for the available data we have analyzed, the present gene had its expression down-regulated under *Fusarium* infection (at 50 h after inoculation), which implies that more studies are needed to understand the specific mode-of-action of this gene. In fact, the advance in the identification of the genes behind QTL and the understanding of their mechanisms for FHB resistance in wheat are still in the initial steps, however, important projects are underway (Steiner et al., 2017).

In this study we have applied an entirely *in silico* approach aiming to unveil the genetic architecture of FHB resistance in bread wheat, joining the available QTL data and genomic resources of the cereal. Successfully, we could assembly an important part of the promised “whole puzzle” of FHB resistance, from QTL to differentially expressed genes, using only publicly available data. It is important to emphasize that after any *in silico* investigation of this kind, the confirmation of the involvement of any of these loci, especially the candidate genes listed, in FHB resistance, can only be performed through further methods, such as gene cloning, gene silencing, and even other more recent methods, such as the metabolo-transcriptomics/proteomics that have already been performed for *Fhb1* and *Fhb2* loci (Gunnaiah et al., 2012; Dhokane et al., 2016; Rawat et al., 2016). The results obtained through this study can support the improvement in breeding strategies for FHB resistance, through biotechnological techniques such as gene-editing of target genes (Bortesi and Fischer, 2015). Our findings can also help to drive a more precise marker assisted selection for these loci. Finally, these results might contribute for the better understanding of the mechanisms related to FHB resistance in wheat.

## Author Contributions

AC, EV, RS, and DF contributed for the conception and design of the study. EV organized the database and performed all data analysis and wrote the first draft of the manuscript. VR contributed for the database organization. EV, RS, LM, CP, and AC wrote sections of the manuscript. AC performed the final revision of the manuscript. All authors contributed to manuscript revision, read, and approved the submitted version.

## Conflict of Interest Statement

The authors declare that the research was conducted in the absence of any commercial or financial relationships that could be construed as a potential conflict of interest.

## Supplementary Material

Supplementary File 1 Database (QTL data) collected for the study.

Supplementary File 2 Maps and QTL files prepared to run the meta-analysis performed through this study.

Supplementary File 3 Meta-QTL generated positioned onto the consensus map.

Supplementary File 4 Candidate genes mined within meta-QTL 1/3B.
